# SLFN11 can sensitize tumor cells towards IFN-γ-mediated T cell killing

**DOI:** 10.1371/journal.pone.0212053

**Published:** 2019-02-12

**Authors:** Riccardo Mezzadra, Marjolein de Bruijn, Lucas T. Jae, Raquel Gomez-Eerland, Anja Duursma, Ferenc A. Scheeren, Thijn R. Brummelkamp, Ton N. Schumacher

**Affiliations:** 1 Division of Molecular Oncology & Immunology, Oncode Institute, The Netherlands Cancer Institute, Amsterdam, The Netherlands; 2 Division of Biochemistry, Oncode Institute, The Netherlands Cancer Institute, Amsterdam, The Netherlands; 3 Division of Cell Biology, The Netherlands Cancer Institute, Amsterdam, The Netherlands; 4 CeMM Research Center for Molecular Medicine of the Austrian Academy of Sciences, Vienna, Austria; 5 Cancergenomics.nl, Amsterdam, The Netherlands; Universita degli Studi di Palermo, ITALY

## Abstract

Experimental and clinical observations have highlighted the role of cytotoxic T cells in human tumor control. However, the parameters that control tumor cell sensitivity to T cell attack remain incompletely understood. To identify modulators of tumor cell sensitivity to T cell effector mechanisms, we performed a whole genome haploid screen in HAP1 cells. Selection of tumor cells by exposure to tumor-specific T cells identified components of the interferon-γ (IFN-γ) receptor (IFNGR) signaling pathway, and tumor cell killing by cytotoxic T cells was shown to be in large part mediated by the pro-apoptotic effects of IFN-γ. Notably, we identified schlafen 11 (SLFN11), a known modulator of DNA damage toxicity, as a regulator of tumor cell sensitivity to T cell-secreted IFN-γ. SLFN11 does not influence IFNGR signaling, but couples IFNGR signaling to the induction of the DNA damage response (DDR) in a context dependent fashion. In line with this role of SLFN11, loss of SLFN11 can reduce IFN-γ mediated toxicity. Collectively, our data indicate that SLFN11 can couple IFN-γ exposure of tumor cells to DDR and cellular apoptosis. Future work should reveal the mechanistic basis for the link between IFNGR signaling and DNA damage response, and identify tumor cell types in which SLFN11 contributes to the anti-tumor activity of T cells.

## Introduction

Immunotherapeutic approaches are emerging as a revolutionary class of cancer therapeutics with clinical benefits across a series of cancer types. Specifically, infusion of antibodies blocking the action of the T cell inhibitory molecules CTLA-4 and PD-1 has shown clinical benefit in, amongst others, melanoma, non-small cell lung cancer, and urothelial carcinoma [1,2]. Furthermore, direct evidence for T cell-mediated tumor regression comes from adoptive T cell transfer studies using tumor-infiltrating lymphocytes (TIL) for melanoma [3], and chimeric antigen receptor (CAR)-modified T cells for B cell malignancies [4]. Despite these impressive clinical results, a large fraction of patients does not benefit from current immunotherapies and relapses are common, motivating a search for mechanisms that influence tumor cell sensitivity to T cell effector mechanisms. In recent work, selection of inactivating mutations in genes in the IFNGR signaling pathway and antigen presentation pathway was shown to occur in tumors that relapsed after PD-1 blockade [5]. Likewise, mutations in the IFNGR pathway have been observed in tumors not responding to CTLA-4 [6] and PD-1 [7] blockade. In line with these data, inactivation of components of the IFNGR pathway and antigen presentation machinery were identified in recent CRISPR-based genetic screens aimed at the unbiased exploration of tumor cell resistance mechanisms towards T cell attack [8–11]. The loss of components of the antigen presentation machinery is readily explained by the selective survival of tumor cells that no longer present T cell-recognized antigens. However, loss of components of the IFNGR signaling pathway may be explained in different ways. First, by modulating the expression of genes in the antigen processing and antigen presentation pathway, impaired IFNGR signaling may reduce presentation of tumor antigens [12]. Second, IFN-γ has also been shown to have direct cytopathic effects on a subset of human cells, but mechanisms that lead to this effect have only partly been elucidated [13].

In this study, we performed a haploid genetic screen to identify tumor cell resistance mechanisms to T cell killing. Using this approach, we identified the direct cytotoxic effect of IFN-γ as a major effector mechanism of T cells in this system. Surprisingly, we identified SLFN11, an IFN-inducible gene previously shown to influence tumor cell sensitivity to DNA damaging agents (DDA), as a modulator of HAP1 sensitivity to T cell attack [14,15]. Notably, interference with SLFN11 expression reduced sensitivity of HAP1 to both IFN-γ and DNA damaging agents. In contrast, in cell lines that showed a much lower sensitivity to IFN-γ-induced cell death, interference with SLFN11 expression reduced their sensitivity to DNA damaging agents but not IFN-γ. Evidence for a link between IFNGR signaling and DDR was provided by the observation of IFN-γ-induced phosphorylation of H2AX. Collectively, our data reveal an unexpected link between a known DNA damage response modulator and sensitivity of tumor cells to cytotoxic T cell attack.

## Results

### A haploid genetic screen for resistance mechanisms to T cell killing

**Fig 1:**
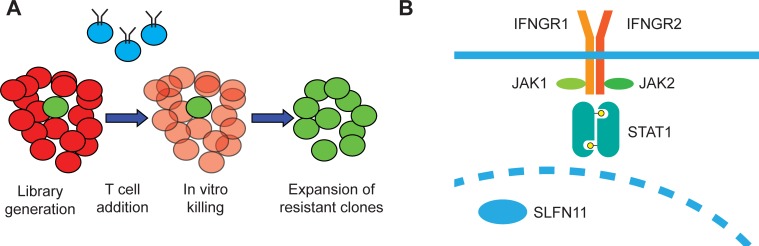
A haploid genetic screen for resistance to T cell mediated killing. A) Experimental scheme: A library of gene trap-mutagenized haploid HAP1 cells was generated, in which most cells harbor irrelevant mutations (red) and rare cells harbor mutations that reduce their sensitivity towards T cell killing (green). Upon exposure to T cells (depicted in blue), cells with reduced sensitivity are positively selected and, following expansion, integration sites in the surviving cell population are analyzed. B) List of most significantly enriched hits. Yellow: components of the IFNGR signaling pathway; green: SLFN11.

In order to identify cancer cell-intrinsic mechanisms of resistance to T cell-mediated cytotoxicity, we set up a whole-genome loss-of-function haploid cell screen. To generate a system in which tumor cells can be exposed to defined T cell pressure, the HLA-A2-positive haploid human cell line HAP1 [16] was modified to express the HLA-A2-restricted MART-1_(26–35, 27 A>L)_ epitope. Subsequently, a library of loss-of-function mutant cells was generated by transduction with a gene-trap vector, and this mutant cell library was exposed to T cells transduced with the MART-1_(26–35, 27 A>L)_-specific 1D3 TCR [17]. After 24h of co-culture, T cells were removed and surviving cells were expanded. Subsequently, gene-trap vector integration sites were sequenced in order to identify genes whose loss conferred resistance to T cell attack, as depicted in Fig 1A. In line with recent data obtained in patients developing resistance to T cell checkpoint blockade and data obtained in CRISPR-based in vitro and in vivo screens [5–11], all canonical components of the IFNGR pathway [18] were identified as dominant hits. In addition, a single IFN-γ-induced gene was identified as a recurrent integration site in T cell resistant HAP1 cells, the putative DNA/RNA helicase SLFN11 (schematized in Fig 1B [15]). Notably, contrary to other studies, none of the components of the antigen presenting machinery was identified as a dominant hit, suggesting that in this system impaired presentation of antigen by a tumor cell clone forms less of a selective advantage than impaired IFNGR signaling.

### IFN-γ-mediated T cell toxicity is alleviated by SLFN11 loss in HAP1 cells

**Fig 2:**
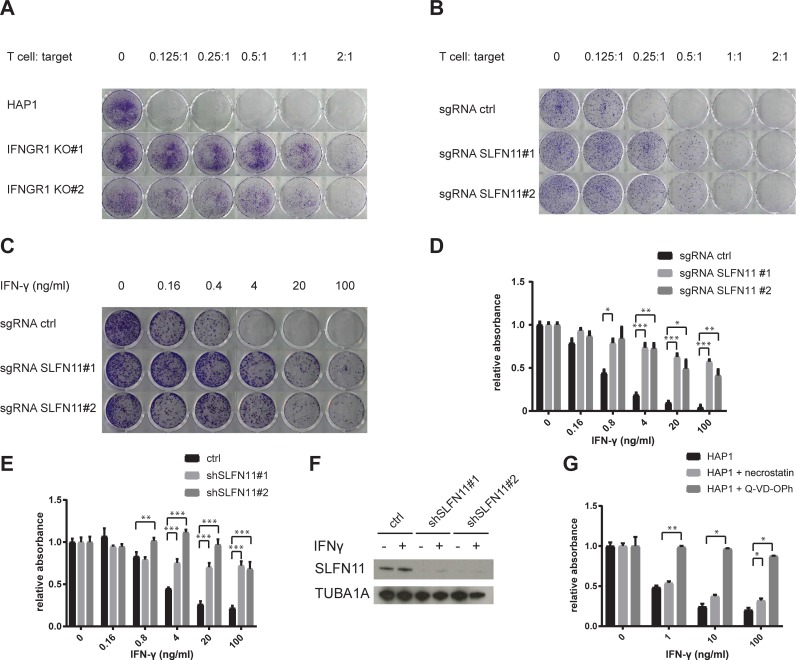
Interference with the IFNGR pathway and SLFN11 protects HAP1 from T cell- and IFNγ-mediated toxicity. A) Parental HAP1 or two IFNGR1 KO clones were exposed to T cells at the indicated effector: target ratio for 24 h. 7 days after T cell exposure, surviving cells were stained with crystal violet. B-C) HAP1 cells transduced with a Cas9-encoding lentiviral vector with either a non-targeting sgRNA (sgRNA ctrl) or two independent sgRNA targeting SLFN11 (sgRNA SLFN11#1 and#2) were exposed to either T cells at the indicated effector: target ratio for 24 h (B), or to IFN-γ at the indicated concentrations for the whole duration of the experiment (C). 7 days after T cell or IFN-γ exposure, surviving cells were stained with crystal violet. D) HAP1 cells transduced with a Cas9-encoding lentiviral vector with either a non-targeting sgRNA (sgRNA ctrl) or two independent sgRNA targeting SLFN11 (sgRNA SLFN11#1 and#2) were exposed to the indicated concentrations of IFN-γ. 48 hours after IFN-γ exposure, cell viability was assayed by analysis of metabolic activity. E) HAP1 cells transduced with a control lentiviral vector or with two independent lentiviral vectors encoding SLFN11-targeting shRNA were exposed to the indicated concentrations of IFN-γ. 48 hours after IFN-γ exposure, cell viability was assayed by analysis of metabolic activity. F) Validation of SLFN11 KD by western blot analysis of untreated or IFN-γ treated (10 ng/ml, 24 h) HAP1 cells. G) HAP1 cells that were either untreated or pre-incubated for 1 h with the indicated compounds (20 μM for necrostatin, 10 μM for Q-VD-OPh) were exposed to the indicated concentrations of IFN-γ. 48 hours after IFN-γ exposure, cell viability was assayed by analysis of metabolic activity. * p<0.05, ** p<0.01, *** p<0.001.

**Fig 3:**
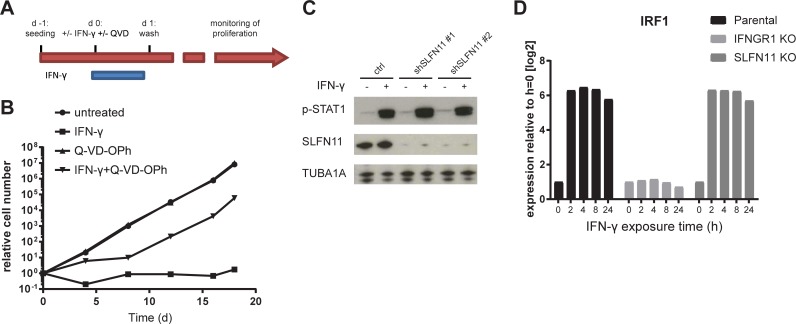
SLFN11 does not regulate IFNGR signaling and evaluation of IFN-γ effects in HAP1. A) Schematic overview of experimental design in B. B) Growth kinetics of cells left untreated or treated with 10 ng/ml of IFN-γ for 24 h, in the presence or absence of Q-VD-OPh. C). Western blot analysis of phosphorylated STAT1 in HAP1 cells transduced with a control lentiviral vector or with lentiviral vectors encoding independent SLFN11-targeting shRNA, either left untreated or exposed to 10 ng/ml of IFN-γ for 24h. D) IRF1 transcript levels following exposure to 10 ng/ml of IFN-γ for the indicated times in parental, IFNGR1 KO, and SLFN11 KO cells.

To directly test the contribution of the IFNGR pathway on T cell mediated killing of HAP1 cells, we generated IFNGR1 KO HAP1 cells using CRISPR-Cas9 and exposed these cells to T cells at different effector: target cell ratios. In line with the results from the haploid genetic screen, HAP1 cell sensitivity to T cells was highly reduced by IFNGR deficiency (Fig 2A). To evaluate whether SLFN11 also influences sensitivity of HAP1 cells towards T cell-mediated toxicity, SLFN11 deficient HAP1 cells were generated and exposed to tumor-specific T cells. Comparison of survival of SLFN11-deficient and -proficient clones demonstrated that the SLFN11 putative DNA/RNA helicase enhances sensitivity of HAP1 cells to T cell-mediated cytotoxicity (Fig 2B). To subsequently assess through which pathway SLFN11 determines the outcome of T cell recognition of tumor cells, we first exposed either SLFN11-proficient or SLFN11-deficient HAP1 cells to IFN-γ. Both in a 7-day clonogenic assay (Fig 2C), and in a short-term cell survival assay (Fig 2D), the dose-dependent toxicity of IFN-γ in HAP1 cells was mitigated by inactivation of SLFN11. By the same token, knockdown (KD) of SLFN11 using shRNA resulted in increased cell survival following IFN-γ exposure (Fig 2E and 2F). To evaluate whether the sensitivity toward T cell cytotoxicity that is conferred by SLFN11 is fully explained by IFN-γ-mediated toxicity, we disrupted SLFN11 in IFNGR1 KO cells. In cells with impaired IFNGR signaling, inactivation of SLFN11 did not influence sensitivity to T cells, indicating that SLFN11 exclusively operates by sensitizing tumor cells toward IFN-γ-mediated toxicity (S1 Fig). SLFN11 has previously been shown to block DNA replication in situations of replication stress [19,20]. However, the substantial drop in cell numbers in a short time frame following IFN-γ exposure of SLFN11-proficient cells was more suggestive of active programmed cell death. To test this, HAP1 cells were exposed to IFN-γ in the presence or absence of either necrostatin, a RIPK1 inhibitor that prevents necroptosis, or Q-VD-OPh, a pan-caspase inhibitor that prevents apoptosis. Whereas necrostatin treatment did not noticeably enhance cell survival upon IFN-γ exposure, Q-VD-OPh almost completely prevented IFN-γ-induced toxicity, indicating that caspase-dependent programmed cell death is a dominant mechanism of IFN-γ in this context (Fig 2G). Subsequently, we tested whether IFN-γ can still impact long-term tumor cell proliferation when apoptosis is blocked. To this purpose, we exposed HAP1 cells to IFN-γ in the presence or absence of Q-VD-OPh for a 24 hour period and evaluated growth kinetics (experimental scheme in Fig 3A). When cells were left untreated or exposed to Q-VD-OPh only, exponential cell expansion was observed. When cells were exposed to IFN-γ for 24 h in the absence of the caspase inhibitor Q-VD-OPh, relative cell expansion at day 18 was approximately 4.6×10^6^ fold lower than for untreated controls. Interestingly, IFN-γ exposure in the presence of caspase blockade resulted in a delay in expansion that lasted for approximately a week after IFN-γ removal, after which cells resumed expansion with a similar kinetic as untreated controls (Fig 3B).

To obtain mechanistic insight into the regulation of IFN-γ sensitivity by SLFN11, we first evaluated whether SLFN11 influences the magnitude of IFNGR signaling. First, analysis of phosphorylated STAT1 in WT and SLFN11 KD HAP1 cells following IFN-γ exposure demonstrated that SLFN11 levels do not detectably influence proximal IFNGR signaling (Fig 3C). As phosphorylation of STAT genes by itself does not necessarily imply IFN-γ -induced transcriptional activity [21], we also exposed parental, SLFN11 KO, or IFNGR1 KO cells to IFN-γ and evaluated the transcriptional induction of a series of IFN-γ inducible genes. As expected, IFNGR deficiency abolished induction of all genes tested (IRF1 (Fig 3D), IDO1, HLA-A, PD-L1 and IFIT3 (S2A–S2D Fig). In contrast, induction of all these genes was fully maintained in SLFN11 KO cells. Consistent with these data, SLFN11 was not a hit in two independent haploid screens for regulators of IRF1 and PD-L1 upon IFN-γ treatment [22,23]. Collectively, these data imply that SLFN11 regulates IFN-γ-mediated toxicity without altering IFNGR signaling activity but rather influences the functional outcome of IFNGR signaling.

### SLFN11 regulates sensitivity toward IFN-γ in a context dependent fashion

**Fig 4:**
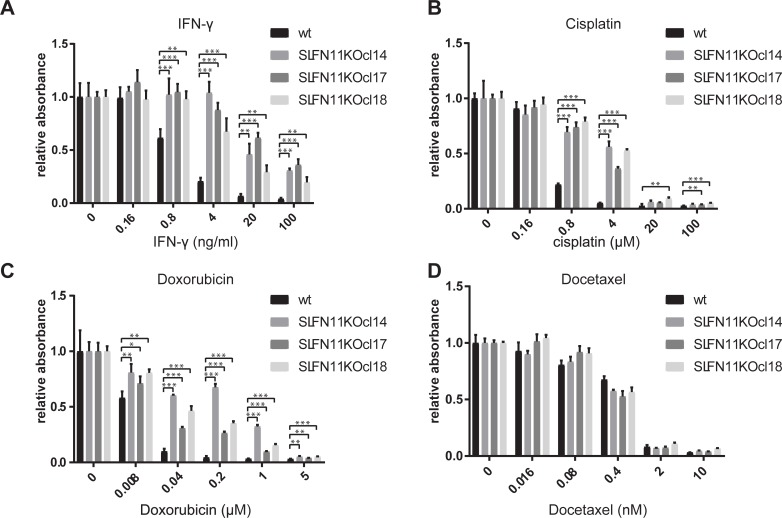
Interference with SLFN11 expression protects HAP1 from IFN-γ and DDA. A-D) Parental HAP1 cells or three SLFN11 KO clones were exposed to the indicated concentrations of IFN-γ (A), cisplatin (B), doxorubicin (C), or docetaxel (D). 48 hours after IFN-γ or chemotherapy exposure, cell viability was assayed by analysis of metabolic activity. * p<0.05, ** p<0.01, *** p<0.001.

SLFN11 has been implicated as a key determinant of tumor cell sensitivity to DNA damaging agents, but not other types of chemotherapeutics [15]. Subsequent work has provided evidence for a role of SLFN11 in blocking replication forks under conditions of DNA replication stress [19,20]. We sought to assess whether the role of SLFN11 as a mediator of T cell- and IFN-γ-induced cell death is linked to its role in mediating cell death induced by DNA damaging agents. We generated SLFN11 deficient HAP1 clones, and exposed these to different types of chemotherapeutic agents or to IFN-γ. As also observed for SLFN11 KD clones, HAP1 clones deficient in SLFN11 showed a decreased sensitivity toward IFN-γ-mediated toxicity (Fig 4A). Furthermore, in line with the data from Pommier and colleagues [15], loss of SLFN11 resulted in decreased sensitivity towards the DNA damaging agents cisplatin and doxorubicin (Fig 4B and 4C), whereas sensitivity to the microtubule disrupting agent docetaxel was unaffected (Fig 4D).

**Fig 5:**
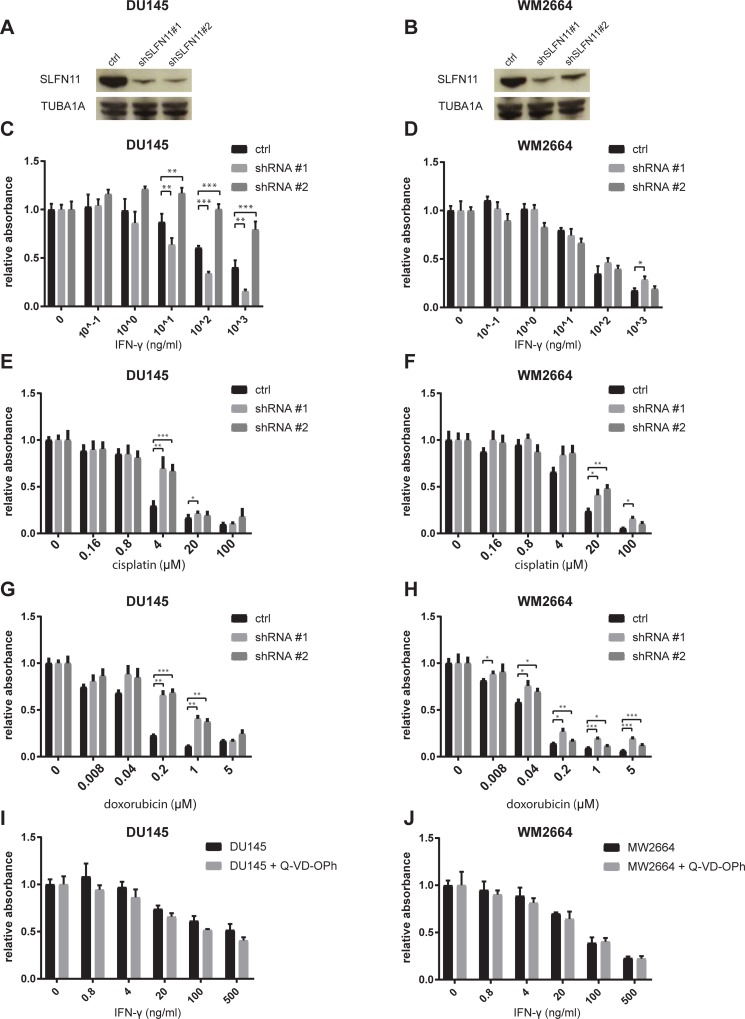
Sensitization of melanoma and prostate cancer cells to DDA but not IFN-γ. A-B) Validation of SLFN11 KD by western blot in DU145 (A) and WM2664 (B). C-D) DU145 (C) and WM2664 cells (D) transduced with a control lentiviral vector or with independent SLFN11-targeting shRNA were exposed to the indicated concentrations of IFN-γ. 7 days after IFN-γ exposure, cell viability was assayed by analysis of metabolic activity. E-H) DU145 (E, G) and WM2664 (F, H) cells transduced with a control lentiviral vector or with independent vectors encoding SLFN11-targeting shRNA were exposed to the indicated concentrations of cisplatin (E, F) or doxorubicin (G, H). 48 hours after exposure, cell viability was assayed by analysis of metabolic activity. I-J) DU145 (I) and WM2664 (J) cells that were either untreated or pre-incubated for 1 h with Q-VD-OPh (10μM) were exposed to the indicated concentrations of IFN-γ. 7 days after IFN-γ exposure, cell viability was assayed by analysis of metabolic activity. * p<0.05, ** p<0.01, *** p<0.001.

**Fig 6:**
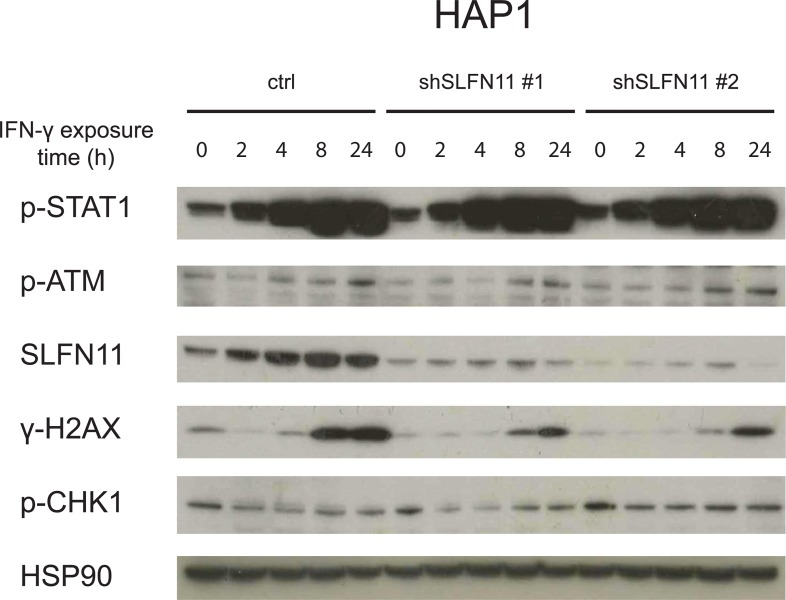
DDR in IFN-γ-treated HAP1. Western blot analysis of the indicated proteins in control or SLFN11 KD HAP1 cells following exposure to 10 ng/ml of IFN-γ for the indicated time periods.

To assess whether SLFN11 influences IFN-γ- and DDA-induced cell death in a parallel manner in different cell systems, we generated SLFN11 KD variants of the prostate cancer cell line DU145 and the melanoma cell line WM2446, chosen because of their high levels of SLFN11 expression. Notably, interference with SLFN11 expression failed to show a consistent protective effect against IFN-γ exposure in these lines (Fig 5A–5D). As in DU145 we observed that one of the shRNAs confer reduced sensitivity and the other increased sensitivity toward IFN-γ, despite similar efficiency in SLFN11 KD, we conclude that those effects are SLFN11-unrelated (Fig 5A–5C). As a control, sensitivity towards both cisplatin and doxorubicin was reduced upon SLFN11 KD in both cell lines, consistent with prior data (Fig 5E–5H). Thus, whereas the effect of SLFN11 on DDA-induced cell death is invariant between these cell systems, the role of SLFN11 in IFN-γ-induced cell death shows context dependency. Lack of SLFN11 involvement in the inhibitory effects of IFN-γ on DU145 and WM2664 may potentially be explained by reliance on distinct pathways of cell inhibition. Indirect support for this notion was provided by the observation that, as compared to the effect on HAP1 cells, IFN-γ-induced inhibition of cell proliferation was a) more modest (see Fig 2D and 2E and Fig 4A for comparison) and b) took substantially longer to manifest (48 hours for HAP1 vs. 7 days for the other lines). To directly test whether IFN-γ inhibits cell expansion by different mechanisms in HAP1 versus DU145 and WM2664, we exposed DU145 and WM2664 to increasing amounts of IFN-γ in the presence or absence of Q-VD-OPh. Contrary to what was observed for HAP1, DU145 and WM2664 cells were not protected from IFN-γ by caspase inhibition (Fig 5I and 5J, see Fig 2G for comparison). Given the role of SLFN11 in sensitization of tumor cells toward DDA-mediated toxicity, we also sought to assess whether there is an association between IFN-γ exposure and induction of a DNA damage response. To this purpose, SLFN11-proficient or -deficient HAP1 cells were exposed to IFN-γ. Within 8 hours following exposure an induction of DDR was observed, as revealed by a minor increase in phosphorylated ATM and a major increase in phosphorylated histone H2AX (γ-H2AX, Fig 6).

### Restoration of a functional SLFN11 locus reverts the phenotype of SLFN11-deficient HAP1 cells

**Fig 7:**
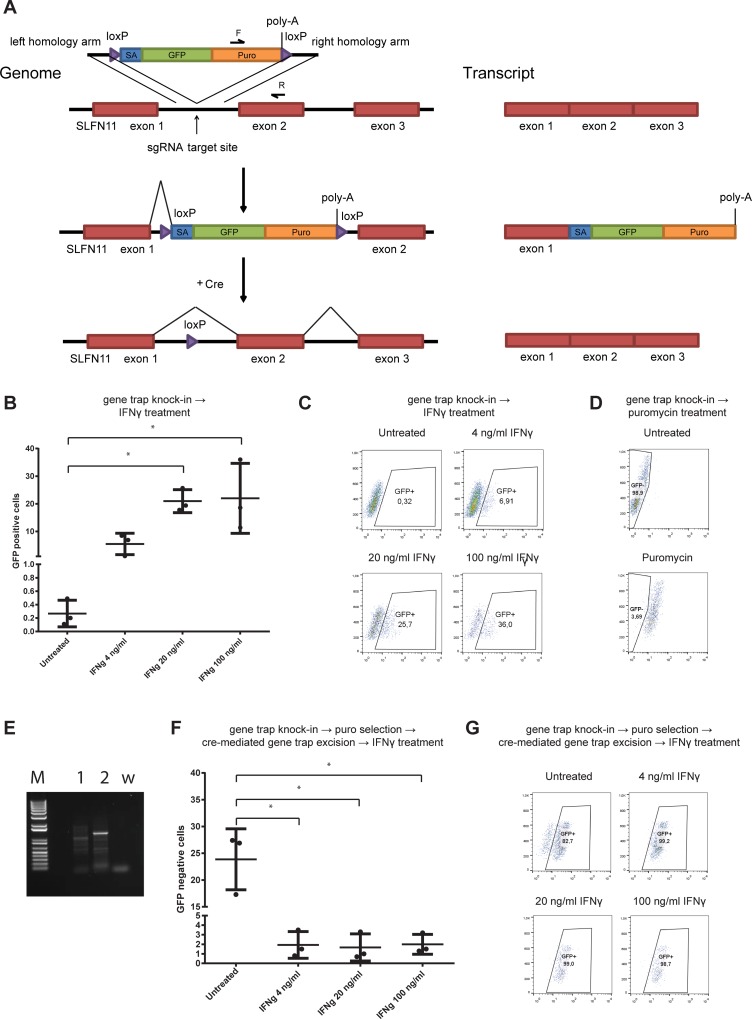
Reversible inhibition of IFN-γ sensitivity by inactivation of the SLFN11 locus. A) Schematic overview of experimental design. A promoterless gene trap vector was inserted into the first intron of the SLFN11 gene. SLFN11 exons are indicated in red, GFP and puromycin N-acetyltransferase coding sequences in green and orange, and loxP sites in purple. Left part of the panel depicts the genomic locus, right part depicts the resulting transcripts. B-C) Cells with integrated gene trap vector were either left untreated or exposed to the indicated IFN-γ concentrations. Technical replicates (B) and representative flow plots (C) are depicted. D) Puromycin treatment results in homogeneous selection of cells with integrated gene trap vector. E) Detection of the integrated gene trap vector in the SLFN11 locus by PCR. Used primers are depicted in panel A (F and R). 1: untransfected HAP1; 2: transfected and puromycin-selected HAP1; w: water control. F-G) Excision of the gene trap vector results in re-sensitization of HAP1 cells to IFN-γ. Cells from panel D were transfected with a cre expressing vector and either left untreated or exposed to the indicated IFN-γ concentrations. Technical replicates (E) and representative flow plots (F) are depicted. * p<0.05.

Genetic complementation of SLFN11 deficient cells with a lentiviral vector expressing SLFN11 cDNA did not revert the phenotype of SLFN11 deficient cells (S3 Fig), either suggesting that SLFN11 function may rely on a distinct transcript, or that transient SLFN11 deficiency may result in a permanent reduction in IFN-γ sensitivity. To address whether renewed expression of the *SLFN11* locus in cells in which the *SLFN11* gene has previously been inactivated restores their sensitivity to IFN-γ, we generated a gene trap vector encoding a GFP-puromycin N-acetyltransferase fusion protein flanked by lox-p sites and homology arms for a genomic region in the first exon of SLFN11. Integration of this vector by CRISPR-mediated knock-in allows disruption of the SLFN11 coding frame, tracking and selection of the gene-modified cells by visualization of GFP and puromycin treatment, respectively, and the reactivation of SLFN11 by excision of the gene trap using Cre recombinase (Fig 7A). We co-transfected HAP1 cells with the vector encoding the excisable gene trap, together with a vector expressing Cas9 and sgRNA targeting the first exon of SLFN11. Notably, subsequent exposure of the transfected cells to IFN-γ resulted in an approximately 100-fold increase in GFP^+^ cells (Fig 7B and 7C), indicating a selective enrichment for SLFN11 deficient cells consistent with our prior observations. In order to assess whether reactivation of the *SLFN11* locus restores sensitivity toward IFN-γ, we first exposed gene trap-transfected HAP1 cells to puromycin, in order to select for those cells in which the gene trap is active, yielding a population of >95% modified cells (Fig 7D). Furthermore, site-specific integration of the gene trap vector was shown by PCR using one primer binding on the gene trap and one primer binding on the SLFN11 genomic region (Fig 7A–7E). After 10 days, transfection of Cre was used to induce excision of the gene trap vector, thereby restoring the *SLFN11* locus in a fraction of the cells. Importantly, exposure of this cell mixture to IFN-γ resulted in the preferential killing of cells with restored SLFN11 expression (Fig 7F and 7G). Thus, IFN-γ sensitivity of HAP1 is a property that toggles with activity of the *SLFN11* gene.

## Discussion

We designed a whole-genome loss-of-function screen to identify mechanisms of tumor cell escape from T cell-mediated toxicity in haploid HAP1 cells (Fig 1A). Similar to what has been observed for CRISPR-based screens in other cell systems [8–11], we identified the IFNGR pathway as a major pathway of tumor cell resistance. In contrast to other studies, no significant enrichment of cells with mutations in components of the antigen presentation pathway was observed (Fig 1B). This observation was surprising, as it suggested that loss of sensitivity to IFN-γ provided a greater selective advantage than impaired antigen presentation. Subsequent experiments indeed demonstrated that, in HAP1, IFN-γ is a major source of toxicity upon T cell recognition, as interference with the IFNGR pathway is strongly protective against T cell-mediated toxicity (Fig 2A). The mechanism of IFN-γ-induced cell death in HAP1 appears distinct from that in other cell systems. First, cell death induction by IFN-γ is observed at >10 fold lower concentrations in HAP1 than in DU145 and WM2664. Second, whereas exposure to IFN-γ mainly results in growth impairment for DU145 and WM2664 (Fig 5C and 5D), it rapidly induces caspase-dependent cell death in HAP1 (Fig 2G). As IFN-γ is not directionally released in the immunological synapse [24], the differential sensitivity of tumors to IFN-γ may determine the relative importance of direct tumor cell killing versus bystander killing in T cell-mediated tumor control, and it will be important to identify characteristics of tumors with IFN-γ hypersensitivity.

SLFN11 was identified as the sole major hit in our screen that was known to be IFN-regulated [25]. SLFN11 is a putative DNA-RNA helicase that has been shown to sensitize tumor cells toward DNA damage-induced cell death [14,15]. In addition, SLFN11 has been implicated in inhibition of HIV protein synthesis in a codon-usage-dependent manner [26]. However, to date SLFN11 expression has not been linked to IFN-γ-induced toxicity or resistance to tumor-specific T cells. Here we demonstrate that SLFN11 expression can regulate IFN-γ-mediated toxicity (Fig 2C–2E, 4A) and, as a consequence, sensitivity of tumor cells to T cell attack (Fig 2B). This role of SLFN11 is independent of regulation of IFNGR signaling, as shown by unaltered induction of a series of IFNGR target genes (Fig 3D and S2 Fig). We observed that in HAP1 SLFN11 also functioned as a modulator of sensitivity towards DDA (Fig 4B and 4C), as previously described in other cell systems [15]. Furthermore, evidence for a possible link between IFNGR signaling and DNA damage response pathways was obtained by the observed induction of a γ-H2AX upon IFN-γ exposure of HAP1 (Fig 6). While the role of SLFN11 as a modulator of sensitivity to DDA was shared in all cell systems analyzed here, the role of SLFN11 as a mediator of IFN-γ-mediated toxicity was specifically observed in HAP1. Future work should reveal the mechanistic basis for the context dependency of SLFN11 as a modulator of IFN-γ but not DDA sensitivity. Related to this, a number of recent studies have demonstrated that the mechanism of action of SLFN11 in DDR response involves recruitment to sites of DNA damage and subsequent blockade of fork progression during replication stress [19,20,27]. It will be of interest to assess whether a similar mode of action is responsible for its role in regulation of IFN-γ-mediated toxicity. A better understanding of the mechanisms that allow SLFN11-mediated toxicity following IFN-γ exposure should be of value to enhance the anti-tumor effect of T and NK cell-based cancer immunotherapies.

## Materials and methods

### Cell lines

HAP1 cells have been described previously [28]. DU145 were a kind gift of J. Neefjes (Leiden University), WM2664 were a kind gift of R. Bernards, and were authenticated by STR from the source. HAP1 cells were maintained in IMDM (Thermo Fisher Scientific) supplemented with 10% fetal calf serum (FCS, Sigma-Aldrich), 100 U ml^−1^ penicillin–streptomycin (Thermo Fisher Scientific) and L-glutamine (Thermo Fisher Scientific); DU145 and WM2664 were maintained in DMEM supplemented with 10% FCS (Sigma-Aldrich) and 100 U ml^−1^ penicillin–streptomycin (Thermo Fisher Scientific). Cells were tested for mycoplasma by PCR. To generate a MART-1_(26–35, 27 A>L)_ epitope expressing HAP1 variant, the coding sequence of MLNA (aa 18–38, 27 A>L) was cloned in front of the coding sequence of the Katushka protein with the two open reading frames linked by P2A coding sequence, and was subcloned into pCDH-CMV-MCS-EF1-copGFP (System Bioscience) using XbaI—SalI sites.

### Generation of MART-1-specific T cells

Retroviral transduction of T cells to generate MART-1-specific T cells has been described previously (17). In brief, retroviral particles were produced by transfecting the pMP71-1D3 vector that encodes the MART-1-specific 1D3 TCR (17) into FLYRD18 packaging cells. Leucocytes were purified from healthy donor buffy coats (Sanquin) using Ficoll density gradients (Sigma-Aldrich), T cells were activated and magnetically isolated using Human T-Activator CD3/CD28 dynabeads (Thermo Fisher Scientific). 48 h after activation, T cells were spin transduced (90’, 2,000 RPM) on retronectin-coated plates (Takara).

### Haploid genetic screen for resistance to T cell pressure

Procedures for the generation of gene-trap retrovirus and HAP1 mutagenesis have been described previously [29]. To select Hap1 variants resistant to T cell pressure, approximatively 10^8^ HAP1 cells (>90% haploid) were exposed to 1D3 transduced T cells for 24 hours, at a ratio of 0.5 TCR transduced T cell/ HAP1 cell). Subsequently, T cells were removed by 3 washes with PBS and surviving HAP1 clones were expanded for 7 days. Integration sites were amplified and analyzed as described in (29).

### Generation of knockout and knockdown cell lines

Knockout cell lines were generated using the CRISPR–Cas9 system. To generate bulk knockout HAP1 cells, cells were transduced with pLentiCRISPR v.2 vector (Addgene 52961) encoding two independent sgRNAs targeting SLFN11. 48 h after transduction, cells were selected with puromycin (2μg ml−1, for two days). In order to generate knockout clones, cells were transfected with px459 vectors (Addgene 48139) encoding either sgRNAs targeting SLFN11 or IFNGR1. Following puromycin selection (2μg ml−1, for two days), single-cell clones were expanded and gene disruptions were validated by sequence analysis and western blot analysis (SLFN11), or by flow cytometry (IFNGR1). The sgRNA sequence acatgaaccctatcgtatat was used for generating IFNGR1 KO clones. The sgRNA sequences tgtcagctgagtctatctag (sgRNA SLFN11#1) and tacactggtctgctaagggg (sgRNA SLFN11#2) were used to generate bulk populations of SLFN11 KO cells. The sgRNA sequence acggaggctaagcgtcgcaa (sgRNA ctrl) served as non-targeting control. Lentiviral shRNA vectors were retrieved from the arrayed TRC human genome-wide shRNA collection. Additional information is available at http://www.broadinstitute.org/rnai/public/clone/search using the TRCN number. The following lentiviral shRNA vectors were used: TRCN0000152057 (shSLFN11#1) and TRCN0000148990 (shSLFN11#2). For production of lentiviral particles, indicated plasmids were co-transfected into HEK293T cells along with packaging plasmids (pPAX2, pVSV-G). Two days after transduction, transduced cells were selected by exposure to puromycin.

### Excisable gene trap

The vector for the excisable gene trap experiment was custom synthesized by Thermo Fisher Scientific. The left homology arm spanned genomic region 35370581–35371183 and right homology arm spanned genomic region 35369965–35370580 of chromosome 17 (assembly Dec.2013 GRCh38/hg38). In between these homology arms, a loxP site, a splicing acceptor sequence, a codon optimized GFP-puromycin N-acetyltransferase fusion protein, an SV40 polyA site, and another loxP site were inserted in this order. To introduce this DNA segment into the SLFN11 locus, >90% haploid HAP1 cells were transfected together with the vector px458 (addgene 48138) expressing the sgRNA agttatctggtatagtcttt, designed such that the majority of the genomic sequence recognized by the sgRNA is located on one of the homology arms and the PAM on the other, in order to avoid Cas9 activity against the transfected plasmid or after integration of the DNA segment.

### MTT and colony forming assays

For experiments involving IFN-γ or chemotherapeutics, 5,000 HAP1/well or 500 DU145 or WM2664/well were plated in 100 μl/well in 96 well plates 24 hours before addition of IFN-γ or chemotherapeutics. Compounds were added in 100 μl of medium to reach the indicated final concentration, and cell viability was assessed after either 2 days (chemotherapeutics or IFN-γ exposed HAP1), or 7 days (IFN-γ exposed DU145 and WM2664). To assess cell viability, supernatants were discarded and cells were incubated with 50 μl of 2.4 mM MTT 3-(4,5-dimethylthiazol-2-yl)-2,5-diphenyltetrazolium (Thermo Fisher Scientific) for 30’ at 37° C. Subsequently, supernatant was removed and cells were incubated in DMSO at room temperature for 15’. 540 nm absorbance was used as a measure of cell viability. For colony forming assays, 25,000 cells/ well (6 well plates) or 5,000 cells/ well (24 well plates) were seeded 24 h before addition of T cells or IFN-γ. Cells were exposed to T cells at the indicated ratio for 24 h, or to IFN-γ at the indicated concentration for the entire duration of the experiment. 7 days after T cell or IFN-γ exposure, cells were washed once with PBS, fixed in ice-cold methanol for 15’, and stained with 0.05% (w/v) crystal violet solution in water.

### Western blot analysis

Cell lysates for western blot analysis were prepared by washing cells with PBS and subsequent lysis in RIPA buffer supplemented with freshly added protease inhibitor cocktail (Roche). After incubation on ice for 30 min, cell lysates were centrifuged at 20,000g for 15 min at 4°C. Supernatants were subsequently processed using a Novex NuPAGE Gel Electrophoresis System, according to the manufacturer’s instructions (Thermo Fisher Scientific). The following antibodies were used for western blot analysis: anti-HSP90: H114 (SantaCruz); anti-TUBA1A: 2144s (Cell Signaling Technology); anti-phosphorylated STAT1 7649s (Cell Signaling Technology); anti γ-H2AX 2577s (Cell Signaling Technology); anti-phosphorylated CHK1 12302s (Cell Signaling Technology); anti-phosphorylated ATM 10H11.E12 (Millipore); anti-SLFN11 HPA023030 (Atlas).

### RNA seq and gene expression

Total RNA was extracted using TRIzol reagent (Ambion life technologies) according to the manufacturer’s instructions. Quality and quantity of total RNA was assessed on a 2100 Bioanalyzer using a Nano chip (Agilent). Total RNA samples having RIN values >8 were subjected to library generation. Strand-specific libraries were generated using the TruSeq Stranded mRNA sample preparation kit (Illumina Inc.) according to the manufacturer's instructions. Libraries were sequenced on a HiSeq2500 using V4 chemistry (Illumina Inc.), and reads (65bp single-end), were aligned against the human reference genome (hg38) using TopHat (version 2.1.0), allowing the spanning of exon-exon splice junctions. TopHat was supplied with a known set of gene models (Ensembl version 77). Samples were generated using a stranded library preparation protocol, in which TopHat was guided to use the first-strand as the library-type. Tophat was run with bowtie 1 version 1.0 and the additional parameters “—prefilter-multihits” and “—no coverage”. In order to count the number of reads per gene, a custom script (ItreeCount) was used. This script is based on the same concept as HTSeq-count and has comparable output. ItreeCount generates a list of the total number of uniquely mapped sequencing reads for each gene that is present in the provided Gene Transfer Format (GTF) file.

## Supporting information

S1 Fig SLFN11-mediated sensitization of HAP1 to T cell pressure is dependent on IFNGR signaling. Parental cells, SLFN11 KO cells, two independent IFNGR1 KO clones, or the same IFNGR1 KO clones in which SLFN11 was subsequently disrupted were exposed to T cells at the indicated effector: target ratio. Viability was assessed by analysis of metabolic activity 7 days after T cells exposure. * p<0.05, ** p<0.01.
(EPS)

S2 Fig SLFN11 does not modulate induction of IFNGR-induced genes. A-D) Transcript levels of IDO1 (A), HLA-A (B), PD-L1 (C) and IFIT3 (D) following exposure to 10 ng/ml of IFN-γ for the indicated times in parental, IFNGR1 KO, and SLFN11 KO cells.
(EPS)

S3 Fig Genetic complementation of does not revert the phenotype of SLFN11-deficient cells. Parental cells, SLFN11 KO cells or SLFN11KO cells in which the cDNA of SLFN11 was overexpressed with a lentiviral vector were exposed to different concentration of IFN-γ. 7 days after IFN-γ exposure, surviving cells were stained with crystal violet.
(EPS)
